# Beyond receiving: feedback literacy as a catalyst for resident growth, a scoping review

**DOI:** 10.1080/10872981.2026.2722433

**Published:** 2026-08-25

**Authors:** Alison Freeth, Sandra Carr

**Affiliations:** a School of Allied Health, Health Professions Education, The University of Western, Perth, WA, Australia; b School of Medicine and Public Health, The University of Newcastle, Newcastle, NSW, Australia

**Keywords:** Feedback literacy, feedback, residents, post graduate medical education, medical education

## Abstract

**Introduction:**

Feedback literacy is gaining momentum in health professions education as an important driver of learning. It involves the capacity to interpret, engage with and act on feedback in ways that promote genuine improvement. While strengthening feedback literacy could make feedback more impactful, it is unclear how effectively resident doctors use these skills in practice.

**Methods:**

This scoping review examines the current landscape of feedback literacy skills among residents through the lens of Carless and Boud's feedback literacy framework of *appreciating feedback*, *making judgements, managing affect* and *taking action*. Using the Joanna Briggs Institute methodology, three databases (MEDLINE, Embase and PsychINFO) were searched for relevant peer-reviewed research studies published between 2015 and 2024. Studies were included that explored residents' feedback literacy in the context of receiving feedback from senior clinicians. Information was extracted for thematic analysis.

**Results:**

Of 1048 articles screened, 15 met inclusion criteria and only one was an intervention designed to enhance feedback literacy. Although residents valued feedback, their engagement with it varied widely. Few studies explored how residents judged their own performance, managed emotional responses or acted on feedback. No studies assessed the long-term impact of feedback literacy training on learning or clinical performance.

**Conclusion:**

Feedback literacy offers a promising pathway to strengthen residents' capacity as purposeful, growth-oriented learners. However, meaningful progress will depend on moving beyond delivering feedback well to explicitly teaching residents how to use it.

## Background

Feedback is one of the most powerful influences on learning [1]. When done well, it helps learners to recognise strengths, identify areas for improvement and refine skills through iterative practice and self-monitoring [2]. Yet junior health professions learners often report that feedback falls short of their needs [3–6]. Early approaches to improving feedback were largely teacher-centric, focusing on the educator's role in effectively delivering information to close the gap between actual and desired performance [7]. More recent work has shifted towards learner-centred perspectives that conceptualise feedback as a dynamic, dialogic process in which learners play an active role [8]. Grounded in sociocultural and social constructivist theories, models such as feedback literacy [8], the educational alliance [9] and the R2C2 model [10] position learners at the centre of a collaborative process that emphasises their own ability to interpret, evaluate and use feedback to guide improvement [11].

Among various approaches, feedback literacy stands out for its focus on the learner's skills and behaviours. Defined as the process by which learners engage with, interpret and respond to information to enhance their work [8], feedback literacy goes beyond merely receiving information. It involves the strategies learners use to make sense of feedback and apply it to their learning [8,12]. It encompasses psychosocial skills, including reflective abilities, openness to critical feedback and making changes to one's behaviour [13]. These skills help learners move beyond passively receiving feedback to actively using it to drive learning.

Feedback literacy was first introduced in the higher education context [14] and then further developed by several scholars [7,8,15,16]. In 2018, Carless and Boud [8] proposed a four-element framework (see Table 1) that has become widely used in feedback literacy research [17] and forms the basis for this review. The first element, *appreciating feedback*, refers to the learner recognising they have an active role in feedback and that feedback is valuable for their learning. The second element, *making judgements*, involves developing accurate self-evaluation skills. The third element, *managing affect*, refers to recognising and regulating the emotions that arise during feedback, particularly navigating defensive reactions to negative feedback. The final element, *taking action*, emphasises acting on feedback and developing strategies for continued learning and improvement. This framework offers a practical structure for understanding and developing feedback literacy skills among learners and educators.

**Table 1.** Elements of feedback literacy framework.

Framework element	Description
Appreciating feedback	What is feedback? Is this feedback helpful for my learning? What does it tell me about my progress?
Making judgements	How am I doing compared to expected standards? Am I making an accurate assessment of my work?
Managing affect	How does this feedback make me feel? How do I manage the feelings that arise during feedback?
Taking action	Am I going to use or ignore this feedback? How do I use this to improve my skills and work?

Description of feedback literacy according to Carless and Boud's framework [8,18].

Feedback literacy is particularly important for junior healthcare learners, including resident doctors. During training, resident doctors rotate through a series of supervised clinical placements to develop the competencies required for independent practice. These environments are complex, requiring residents to balance learning with the delivery of high-quality patient care. Multiple supervising clinicians, variable teaching quality and competing time pressures can limit the effectiveness of feedback [12]. Feedback may also be low in quality [3,6], vague [4,5] or lack clear guidance for improvement [3,4]. Developing feedback literacy may help residents to derive greater value from feedback, even when its quality or availability is limited. Greater attention to feedback literacy may support residents to engage more actively with feedback and translate it into meaningful improvement throughout residency and their professional careers.

Early studies suggest that targeted interventions can strengthen feedback literacy skills [19–21], however the evidence for the methods used and outcomes of this training is still emerging. While existing reviews have explored feedback literacy in higher education [19,22], feedback literacy instruments [23] and medical students' receptiveness to feedback [24], little is known about how feedback literacy has been conceptualised, taught and applied among resident doctors. Mapping this evidence is an important step towards understanding how residents develop and use feedback literacy, and identifying opportunities to better support learning from feedback.

The primary aim of this review was to provide an overview of the existing literature on the feedback literacy skills of resident doctors through the lens of Carless and Boud's feedback literacy framework of *appreciating feedback*, *making judgements*, *managing affect* and *taking action* [8]. A secondary aim was to identify additional factors beyond these four elements that influence how residents engage with and respond to feedback from more senior doctors.

## Methods

The methodology of this scoping review adhered to the Joanna Briggs Institute (JBI) manual for scoping reviews [25] and the Preferred Reporting Items for Systematic Reviews and Meta-Analyses Extension for Scoping Reviews (PRISMA-ScR) checklist [26]. A scoping review was selected to map the extent and nature of available evidence on feedback literacy among residents and to identify gaps in knowledge and guide future research [26]. An initial search identified limited articles on this topic, making this research well suited to a scoping review. A review protocol was developed and registered with Open Science Framework (https://doi.org/10.17605/OSF.IO/DKUZP).

### Eligibility criteria

Our study included original research articles on the topic of feedback literacy among residents, published in peer-reviewed English-language journals, between January 2015 and June 2024. This time frame was chosen based on preliminary research indicating that feedback literacy is a relatively recent area of study [24]. The population of interest was resident doctors, defined as medical graduates without specialist qualifications, including interns, residents and registrars of any postgraduate year (PGY) level. Studies were included if residents received feedback from more senior doctors but excluded feedback from other health professionals. Senior doctors were selected as feedback providers because their extensive clinical and educational experience enables them to assess residents' performance against expected standards and provide accurate feedback information. Feedback from peers, allied health professionals and medical students was excluded because the review focused on supervisory feedback from senior physicians, given its distinct role in clinical oversight, competency assessment, entrustment and progression decisions. This also reduced heterogeneity arising from differences in feedback purpose, authority and educational consequences across stakeholder groups. Feedback could be provided informally, such as during routine clinical work, or formally, for example through summative assessments. The concept examined was feedback literacy, which could be explicitly stated, or implied. Studies were considered to imply feedback literacy when residents demonstrated one or more of Carless and Boud's [8] four elements of feedback literacy (see Table 1), without explicitly using the framework or the term ‘feedback literacy’. This broad definition of feedback literacy allowed for a richer exploration of the topic, acknowledging the varied terminology used by researchers.

### Search strategy

A preliminary search was conducted in MEDLINE to identify articles relevant to the topics of ‘feedback literacy’ and ‘residents’. The key words and index terms (see supplementary file 1) were identified and used to develop a full search strategy with the assistance of a senior health librarian. The search was adapted for three databases, MEDLINE, Embase and PsychINFO (see supplementary file 1) and executed on the 9th of June 2024. No additional articles were identified by a further search on the 12th of December 2024, and no articles were identified from a hand search of the reference lists of included articles.

Retrieved citations were imported into Covidence systematic review software [27] and duplicates removed automatically. The title and abstract screen and full-text review were conducted by two independent reviewers according to the inclusion and exclusion criteria. AF screened all articles and SC and TC independently screened approximately half of the articles each. The full-text review was completed by AF and SC. Regular meetings were held by the research team and conflicts were resolved by consensus.

A data charting form was created in Covidence. It was pilot tested on four articles, after which modifications were made (AF, SC). AF then used the form to extract data from all articles, with further minor revisions to the template made iteratively during the charting process. The extracted information included the author, year of publication, country of origin, aims, description of the study, number of participants, the study method, data analysis approach, description of feedback literacy elements, response to positive and negative feedback and gaps identified by the research.

### Data analysis

The collected data was analysed using thematic and numerical analyses, as recommended for a scoping review [28]. Thematic analysis involves examining excerpts of text to identify, analyse and report patterns, known as themes [29]. In this review, text copied from the included papers was exported from Covidence into an Excel spreadsheet. The text was grouped under predefined headings including the study characteristics, participants, methodology, data analysis, feedback characteristics, feedback literacy elements according to Carless and Boud's [8] framework and factors influencing residents' responses to feedback. Feedback seeking was briefly addressed under *managing affect* in the original framework, however we found it encompassed distinct skills beyond emotion management and therefore it is reported separately to the four elements. Excerpts of extracted text were colour coded, categorised and reviewed in detail to identify patterns among the text using an inductive approach. A list of themes was generated and modified iteratively during the data analysis phase by AF and SC.

Results relevant to the review questions are presented in narrative form and statistical results are presented in tables and graphs. The median number of participants was calculated using Excel. Given this is a scoping review where the goal is to map the available evidence on a topic [25], an appraisal of the quality of evidence was not undertaken.

## Results

### Overview

**Figure 1.**
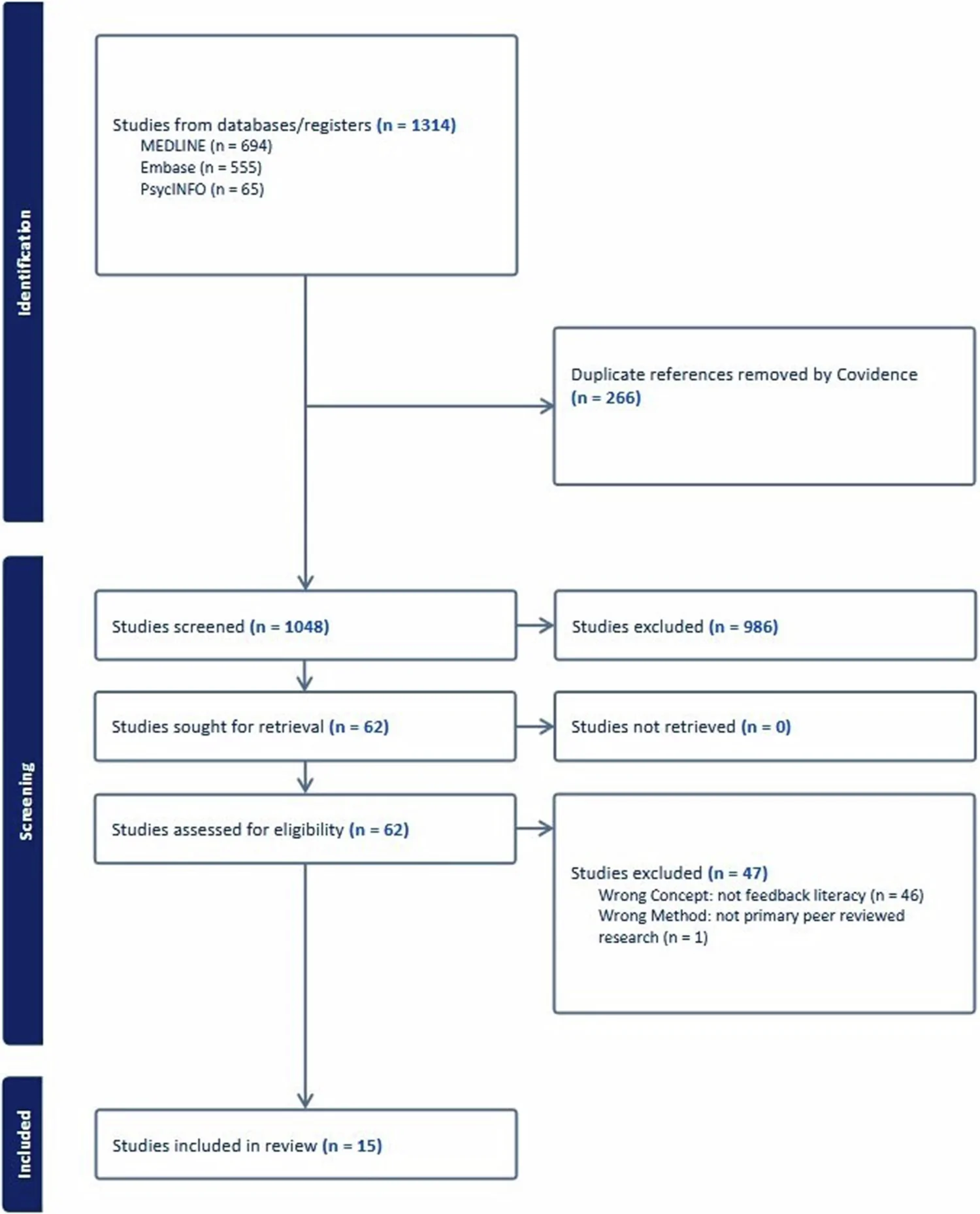
PRISMA flow diagram.

A total of 1314 studies were retrieved and 266 duplicates were removed from the search executed on the 9^th^ of June 2024. The full text was reviewed for 62 articles and 15 studies met the inclusion criteria for the final analysis. A list of the included studies is provided in supplementary file 2. The search outcomes are depicted in Figure 1.

### Characteristics of the articles

Most years in the study period, spanning from 2015 to 2024, had one or two publications. One study was located in Iran [30] and all other studies occurred in Western countries: United States of America *n* = 6 [31–36], Canada *n* = 4 [5,37–39], Australia *n* = 3 [12,40,41] and the United Kingdom *n* = 1 [42].

A qualitative research methodology was used in 14 studies, using focus groups or interviews for data collection (Table 1). One study used a mixed methods approach [30]. This cross-sectional study surveyed 180 residents using a validated 15 item scale and an open-ended question to explore participants attitudes and perceptions of feedback [30].

Six studies incorporated an intervention, programme or tool to improve feedback [5,12,32,36,38,39]. The study characteristics are further detailed in supplementary file 3. Two studies paired a resident with a supervisor via a long term feedback programme [36,39], two used supervisor focused training or tools to improve feedback delivery [5,38], one programme was designed to improve the feedback literacy of residents [12], and one study used a written feedback card with a rating scale and feedback prompts [32]. Of the included studies, 11 were published in journals that focused on education and training, as shown in Table 2.

**Table 2.** Characteristics of the included studies by methods, journal name and resident specialty.

Methodology	Number of studies	Journal of publication	Number of studies
Qualitative	14	Medical Education	4
Thematic Analysis	7	Journal of Graduate Medical Education	2
Grounded Theory	5	Academic Medicine	2
Interpretive phenomenological analysis	1	BMC Medical Education	2
Not specified	1	AEM Education and Training	2
Mixed methods	1	Teaching and Learning in Medicine	1
		Canadian Journal of Emergency Medicine	1
		Neurosurg focus	1
Data collection method		Specialty	
Interview	8	Mixed specialities	5
Focus group	5	Internal medicine	3
Focus group and interview	1	Emergency	2
Questionnaire	1	Psychiatry	2
		Surgery	2
		Anaesthetics	1

### Population and context

A summary of the characteristics of the 15 included studies is provided in supplementary file 3. The number of resident participants ranged from 7 to 180, with a median of 15. All studies were conducted in a clinical setting. Residents ranged in seniority from PGY1 to PGY10 and most studies included residents of multiple training years. The residents were from a range of specialties, as listed in Table 2. Most studies (*n* = 11) involved residents exclusively, while four studies also collected data from their supervisors [34,35,38,39]. For this review, only the resident perspectives were analysed.

### Synthesis of results

#### Feedback characteristics

The feedback characteristics of the included studies are outlined in Table 3. Feedback usually occurred in person. Five studies [5,31,32,36,40] incorporated both written and verbal feedback. Only one study [30] used a validated questionnaire; however, this did not specifically measure feedback literacy. Feedback was examined in both a formal nature, for example through term supervisor meetings [5,34,38], workplace-based assessments [42] and milestone feedback [31] or informally during routine clinical duties and operations [12,32,37,39]. The topic discussed during feedback was rarely reported, except for one study that examined feedback in a surgical context [42].

**Table 3.** Characteristics of feedback.

Feedback characteristics	Number of studies
**Feedback format**	
Verbal	6
Mixed (verbal and written)	5
Not specified	4
**Feedback event**	
Formal feedback (end of term, mid-term, summative assessment)	5
Mixed	2
Informal feedback	1
Not specified	7
**Person providing feedback**	
Term supervisor	7
Consultant (not supervisor)	3
Mixed	3
Not specified	2

#### Feedback literacy

The main aim of this review was to provide an overview of the feedback literacy skills of resident doctors according to Carless and Boud [8] framework of *appreciating feedback*, *making judgements*, *managing affect* and *taking action*. Only one study [12] used the term ‘feedback literacy’. The remaining 14 studies discussed one or more elements of the framework, but without explicitly referring to feedback literacy or citing Carless and Boud's [8] framework. Four studies [32,33,35,38] discussed only one element of the framework, three studies [34,40,41] discussed two elements, eight studies [5,12,30,31,36,37,39,42] discussed three elements and no studies examined all four elements. Overall, *appreciating feedback* was the most discussed element (*n* = 12 studies) and *making judgements* was the least discussed (*n* = 2 studies [12,37]), with 10 studies each discussing *managing affect* or *taking action*. Further detail on the framework elements addressed by each article is included in supplementary file 3.

The study that explicitly examined feedback literacy was a year-long designed-based research study by Noble et al. [12], involving emergency department residents across five clinical rotations. Following a workshop and clinical placement, residents who adopted an agentic approach reported more meaningful feedback interactions. They used strategies such as initiating and guiding feedback conversations, active listening and questioning supervisors. However, their engagement was shaped by factors such as emotional state, workload, supervisor behaviours and departmental factors. Noble et al. [12] concluded that feedback literacy is shaped by context and further research was recommended to explore feedback literacy across different settings and experience levels.

While most studies in this review did not address feedback literacy as an explicit concept, the elements of Carless and Boud's [8] framework were examined across the included studies.

#### Appreciating feedback

Residents generally recognised the importance of feedback for their learning. In the largest study, Shafian et al. [30] surveyed 180 residents and 77.7% of respondents recognised that feedback improved their clinical performance. In other studies, residents valued high-quality feedback, particularly when it was growth-focused [40], timely [5,36], specific [36,39] and offered clear guidance for improvement [36,39]. Even negative feedback, though often uncomfortable, could be perceived as beneficial for growth when delivered constructively [32,34,42].

Some residents took an active role in feedback [35,41] or became more engaged after a feedback literacy workshop [12]. In contrast, a study of neurosurgical residents found feedback from summative assessments was often unidirectional, vague or inaccurate, leading some participants to prefer formative over summative assessment feedback comments [31]. Similarly, anaesthetic trainees in another study reported that informal feedback was more useful and of higher quality than formal feedback [5]. Both studies [5,31] had small sample sizes (*n* = 10 and *n* = 14 respectively) and should be interpreted with caution, yet highlight residents' concerns about the quality of feedback in summative assessment settings.

#### Making judgements

There was limited information published on how residents assess their own skills and knowledge. One study highlighted that fragmented supervision and varying supervisor perspectives could make self-assessment difficult [12]. Another study [37] suggested that residents may lack the experience needed to evaluate their performance confidently and were hesitant to dismiss supervisor feedback when it contradicted their own views. No studies explored the accuracy or process of self-assessment or examined its impact on learning outcomes.

#### Managing affect

Residents experienced a range of emotional responses to feedback, both positive and negative. Some studies linked positive feedback to increased confidence [30,39], but residents more often focused on the negative emotions associated with feedback. Anxiety before scheduled feedback sessions was common, often stemming from the fear of discussing one's weaknesses [36,39,42]. Negative feedback triggered emotions such as shame, defensiveness, stress and humiliation, which could undermine feedback acceptance [5,30,40]. While most studies acknowledged the emotional impact of feedback, there was little guidance on how to manage these emotions. Only Nobel et al. [12] addressed this, inviting residents to share emotionally charged feedback experiences and discuss strategies to regulate emotions during their feedback literacy workshop. However, the outcomes of emotion management training were not reported, nor were they explored in any other studies.

#### Taking action

Residents found action plans most helpful when they were specific, actionable and timely [5,31,36,39], as this facilitated more immediate and intentional behavioural change [31,36,40]. In contrast, some action plans were unclear or lacked detail [5,30,34]. Comments such as ‘needs more experience’ [5] caused frustration, highlighting residents' preference for constructive and actionable feedback [5,42]. Action plans could be resident led, supervisor initiated or co-constructed, though there were no reflections on which approach was most beneficial. Some residents reported developing clearer action plans after a feedback literacy workshop, yet vague responses from supervisors still left others uncertain about how to improve [12]. No studies assessed the long-term implementation of action plans or their impact on clinical practice.

### Other factors that shaped resident feedback responses

The second aim of this review was to identify factors beyond these four elements that influenced how residents engage with and respond to feedback from senior clinicians. The response to feedback was shaped by the quality of the teacher-learner relationship, the interplay between feedback and assessment, the content of feedback and the practice of feedback seeking.

Strong teacher-learner relationships were central to effective feedback. Long-term, supportive educational relationships fostered trust, encouraged open dialogue and enabled more personalised feedback information [34,36,39]. In supportive relationships, residents typically felt safer to share their opinions and were more receptive to feedback, even when they were initially sceptical [5,41]. Telio et al. [41] further suggested that the emotional impact of feedback might be more strongly influenced by the quality of the relationship with the feedback provider than by whether feedback itself was positive or negative.

Feedback and assessment were closely intertwined in shaping residents' responses. When supervisors also served as assessors, some residents were hesitant to reveal weaknesses, fearing negative consequences for their future assessment outcomes [33,40,42]. Feedback was also seen as more meaningful when separated from assessment [39] and framed as a learning opportunity rather than evaluative judgement [42].

The content of feedback also played a significant role in how it was received. Positive feedback was appreciated when it reinforced strengths and encouraged skill development [31,39]. Negative feedback was more likely to be dismissed, especially when delivered within a weak teacher-learner relationship or it lacked credibility [31,34,36]. While residents appreciated praise, many preferred constructive feedback that pinpointed areas for improvement [34,39–42]. Feedback that included growth-oriented comments [5], specific examples of behaviour [34] and was delivered within a supportive relationship [41] was especially valued.

Feedback seeking was also explored in several studies. It was a nuanced process, with residents weighing factors such as their performance [42], confidence with a skill [40], emotional readiness [12] and supervisor characteristics [12,32] before initiating feedback conversations. Barriers included feeling overwhelmed [35], a reluctance to receive negative feedback [32,35,41], time constraints [30,32] and the absence of a workplace culture that encouraged feedback seeking [30].

## Discussion

This scoping review identified 15 studies published between 2015 and 2024 that described feedback literacy, or its concepts, among residents. Across these studies, residents consistently recognised the value of feedback, yet their engagement with it varied considerably. Some approached feedback as an opportunity for growth, actively seeking and using feedback, while others remained more passive, limiting their potential to learn from feedback. Importantly, there are gaps in the literature in understanding how residents put feedback literacy skills into practice, particularly when making judgements about their performance, regulating emotional responses and translating feedback into purposeful action.

Although feedback literacy is conceptually well established, evidence of its practical application in clinical settings remains limited. This review found little evidence that residents had been explicitly taught feedback literacy skills or were consistently using them in everyday practice, suggesting a gap in feedback literacy education from undergraduate through to postgraduate training. This aligns with the findings of a large scoping review of medical students [24], where only 4 out of 98 studies on receptiveness to feedback involved feedback literacy interventions. Even when learners are introduced to feedback literacy concepts, they may find it difficult to implement these skills amid the contextual barriers of clinical work [43]. For residents, these barriers may include high workloads [32,40], inconsistent access to supervisors [12], conflicting feedback messages [12,44], poorly delivered critical feedback [8] and weak feedback cultures [45]. Future educational strategies should therefore consider how feedback literacy can be enacted within the realities of clinical training, providing repeated opportunities for residents to engage with authentic workplace feedback, reflect on performance and act on feedback with appropriate guidance. Without such opportunities, feedback literacy risks remaining a theoretical construct rather than a routine part of clinical learning.

### Improving feedback literacy

Research to guide the development of feedback literacy in postgraduate medical education is in its early stages. However, evidence from higher education offers promising approaches that could be adapted for healthcare settings. A scoping review of 16 interventions found that feedback literacy training can shift learners' perceptions, confidence, agency and engagement with feedback [19]. Strategies with varying levels of success include self and peer assessment [46,47], reflective activities [19], an educator toolkit [48], workshops [12,43] and facilitated feedback discussions [19]. Peer feedback has attracted particular interest [49,50] because giving feedback to others may deepen reflection and understanding of the feedback process [19]. However, the transferability of these approaches to healthcare settings may depend, in part, on the workplace cultures in which they are implemented. Feedback culture shapes not only opportunities for feedback, but also what learners perceive as credible, attend to and ultimately act upon [51]. A culture of politeness may also constrain the open feedback conversations important for improvement [35]. Feedback literacy initiatives should therefore extend beyond developing individual capabilities to recognising and supporting the cultural conditions that shape how feedback is put into practice.

Developing feedback seeking skills offers another important pathway for strengthening feedback literacy. By shifting learners from passive recipients to active participants in their own development, feedback seeking promotes agency, self-assessment and the identification of learning needs. It can generate more specific, actionable insights [52], increase satisfaction with feedback and support greater acceptance of feedback [53]. Although Carless and Boud [8] briefly explored feedback seeking within the domain of *managing affect*, our review suggests that it extends beyond emotional readiness to also involve judgements about the value, timing and source of feedback. Learners may seek feedback to better understand their performance, identify opportunities for improvement or confirm existing self-perceptions [54]. In doing so, they must also weigh anticipated benefits against potential risks [55,56] and identify trustworthy and approachable feedback sources [53,57]. These judgements may be particularly important in residency training, where feedback can be inconsistent and is not always proactively offered. Together, these findings suggest that feedback seeking plays a broader role in learners' engagement with feedback than is currently reflected in Carless and Boud [8] framework. As such, feedback seeking may warrant greater attention in both future conceptualisations of feedback literacy and educational interventions designed to support its development.

Meaningful progress in feedback also depends on addressing the relationships that shape how feedback unfolds in clinical practice. Strong teacher–learner relationships characterised by trust, credibility, open dialogue and a commitment to learner growth can increase feedback acceptance and influence whether feedback is interpreted as developmental or threating [41]. Structured feedback approaches, such as Sargent et al.'s R2C2 model [10], can further support feedback literacy by equipping supervisors to promote active learner participation and reflection [58]. Clinical leaders who model receptivity to feedback, integrate reflection into practice [59] and provide regular, timely feedback [60], help create environments though which feedback is expected, valued and routinely used for learning. Feedback literacy is therefore shaped by the teachers who create the conditions for meaningful feedback, making the development of feedback literate supervisors an important complement to resident-focused initiatives.

### Applying Carless and Boud's framework

Carless and Boud's four-element framework of feedback literacy [8] provided a valuable structure for interpreting our findings and for identifying areas where residents' feedback literacy is underdeveloped. Consistent with other studies [23,58], residents generally *appreciated feedback,* recognising its importance for learning. However, appreciation alone does not guarantee meaningful engagement. Our findings on *managing affect* align with prior research showing that positive emotions promote feedback acceptance, while negative emotions can hinder it [44,61–63]. Yet negative feedback can also be a catalyst for growth [34,42], implying that building emotional resilience and strategies to manage negative emotions could improve the overall feedback experience. The element of *making judgements* about one's skills was notably underrepresented, which may reflect the challenges junior doctors face in accurately evaluating their own performance as they develop competence [64]. The final element, *taking action*, includes the decision to thoughtfully accept or reject feedback. When residents dismiss feedback, it is unclear whether this represents confident self-regulation or a missed opportunity for growth. An important direction for future research is to examine how feedback literacy influences these decisions and whether its development is associated with improvements in learning and clinical performance.

### Implications for educators and future research

These findings have several implications for educators and future research. Although assessing the quality of evidence was beyond the scope of this review, the predominance of descriptive observational studies highlights the need for more rigorous research evaluating the long-term impact of feedback literacy interventions on residents' learning and clinical skills. At the individual level, interventions such as workshops could target residents' skills in adopting an active stance in feedback, managing negative emotions, accurately evaluating performance and taking action to improve skills. Ideally, feedback literacy development should begin early in medical training, with structured opportunities to practice these skills within supportive supervisor relationships and positive workplace cultures. Supervisors would also benefit from training in feedback literacy to foster learner reflection, create psychological safety and build strong teacher-learner relationships that promote meaningful feedback engagement. Clarifying the distinction between formative feedback conversations and discussions that contribute to summative assessment may further support more open and authentic feedback dialogue. Finally, feedback seeking is a complex process that has the potential to increase the relevance of feedback, warranting a significant place in future feedback literacy models and interventions.

### Limitations

This review excluded non-English language articles and may therefore have missed relevant sources of information. We only included studies that examined active engagement with feedback, or addressed one or more elements of feedback literacy according to Carless and Boud's [8] framework. We also focused on feedback provided by senior clinicians as they are highly valued sources of feedback [65] and are well positioned to identify residents' learning needs through their clinical and educational experience, although we acknowledge that learning is shaped by feedback from multiple sources. While these inclusion criteria may have limited the number of articles included, our findings are repeatedly represented across the included papers. Finally, most studies were conducted in Western countries, which may introduce a cultural bias in how feedback literacy was described by residents.

### Conclusion

This review found that despite valuing feedback, many residents remain uncertain how to interpret it, regulate their emotional responses or translate insights into action. Developing feedback literacy represents an important opportunity to strengthen residents' engagement with feedback, but translating this potential into practice will require approaches that are adapted to the realities of workplace-based clinical training. The next challenge is to determine how feedback literacy can be cultivated throughout postgraduate training and assess the long-term impact of this training, because feedback should do more than inform; it should transform.

## Supplementary Material

Supplementary file 1 key words and search strategy.docx

Supplementary file 3 characteristics of included studies.xlsx Supplementary file 3 characteristics of included studies.xlsx

Supplementary file 2 list of included studies.xlsx Supplementary file 2 list of included studies.xlsx

## Data Availability

The data underlying this article will be shared on reasonable request to the corresponding author.
