# Supplementary material for: Beyond receiving: feedback literacy as a catalyst for resident growth, a scoping review
Source: Med Educ Online. 2026 Aug 25;31(1):2722433. doi: 10.1080/10872981.2026.2722433 (PMC13520860; doi:10.1080/10872981.2026.2722433)
Supplement: Supplementary file 1 [file ZMEO_A_2722433_SM2538.docx]

# Supplementary file 1: Key words and search strategy

Key Search Concepts:

|  | Concept 1 | Concept 2 |
| --- | --- | --- |
| Key terms | Feedback literacy | Residents |
| Synonyms | Feedback  Constructive feedback  Formative feedback  Receptivity to feedback  Evaluating feedback  Judging feedback  Attitude to feedback | Intern  Resident  Registrar  Junior doctor |

Search strategy for MEDLINE

|  | Search term | Records Retrieved |
| --- | --- | --- |
| 1 | exp Feedback/ | 34925 |
| 2 | exp Formative Feedback/ | 1190 |
| 3 | 1 or 2 | 36042 |
| 4 | exp "Internship and Residency"/ | 62558 |
| 5 | 3 and 4 | 870 |
| 6 | (feedback adj3 (literacy or recogni* or appreciat* or judg* or evaluate or affect or emotion* or act or action or plan or respons* or attitude* or receptiv* or engag* or percept* or seek*)).ti,ab,kw,kf. | 7958 |
| 7 | (resident or residents or registrar* or intern or interns or "junior doctor").ti,ab,kw,kf. | 222495 |
| 8 | 6 and 7 | 317 |
| 9 | 5 or 8 | 1097 |
| 10 | limit 9 to (English language and yr="2015 -Current") | 733 |
| 11 | limit 10 to "review articles" | 23 |
| 12 | limit 10 to (editorial or guideline or letter or newspaper article) | 16 |
| 13 | 10 not (11 or 12) | 694 |
